# Vitexin Suppresses High-Glucose-upregulated Adhesion Molecule Expression in Endothelial Cells through Inhibiting NF-κB Signaling Pathway

**DOI:** 10.1021/acsomega.4c02545

**Published:** 2024-07-22

**Authors:** Pie-Che Chen, Yun-Ching Chang, Kun-Ling Tsai, Cheng Huang Shen, Shin-Da Lee

**Affiliations:** † Department of Urology, Ditmanson Medical Foundation Chiayi Christian Hospital, Chia-Yi 60002, Taiwan; ‡ Chung Jen Junior College of Nursing, Health Science and Management, Chia-Yi 60002, Taiwan; § School of Medicine, College of Medicine, I-Shou University, Kaohsiung 84001, Taiwan; ∥ Department of Physical Therapy, College of Medicine, National Cheng Kung University, Tainan 70101, Taiwan; ⊥ Institute of Allied Health Sciences, College of Medicine, National Cheng Kung University, Tainan 70101, Taiwan; # Department of Urology, Ditmanson Medical Foundation Chiayi Christian Hospital, Chia-Yi 60002, Taiwan; ¶ Department of Biomedical Sciences, National Chung Cheng University, Min Hsiung, Chia-Yi 60002Taiwan; ◆ Department of Physical Therapy, PhD program in Healthcare Science, China Medical University, Taichung 40202, Taiwan

## Abstract

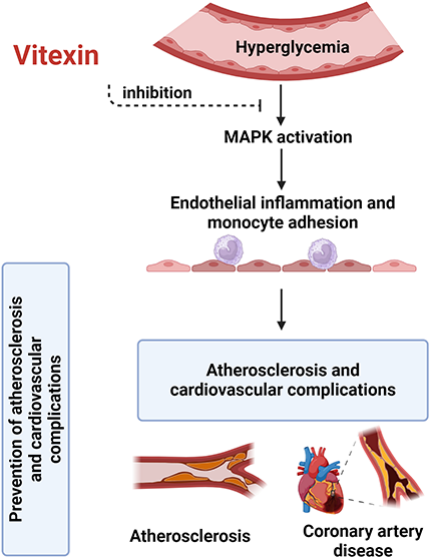

Vascular damage is one of the significant complications of diabetes mellitus (DM). Central to this damage is endothelial damage, especially under high-glucose conditions, which promotes inflammation via the NF-κB signaling pathway. Inflammatory processes in endothelial cells directly contribute to endothelial dysfunction, such as promoting inflammatory cytokine release and activation of adhesion molecules. Vitexin, a compound found in many medicinal plants, shows promise in countering oxidative stress in diabetic contexts and modulating blood glucose. However, its effect on high-glucose-induced endothelial cell activation has not yet been studied. This research explores vitexin’s potential role in this process, focusing on its influence on the NF-κB pathway in endothelial cells. Human umbilical vein endothelial cells (HUVECs) were stimulated with 30 mM glucose (high glucose, HG) with or without vitexin treatment for 24 h. Western blotting assay was conducted for the NF-κB pathway and p-p38. Adhesion molecules (ICAM-1, VCAM-1, E-selectin, and MCP-1) were studied using flow cytometry, while pro-inflammatory cytokines were investigated using ELISA. Monocyte adhesion and vascular permeability tests were conducted to confirm the protective effect of vitexin under HG exposure. This study confirms vitexin’s capacity to suppress p38 MAPK and NF-κB activation under HG conditions, reducing HG-elevated adhesion molecules and pro-inflammatory cytokine secretion. Additionally, vitexin mitigates HG-stimulated vascular permeability and monocyte adhesion. In conclusion, this study shows the therapeutic potential of vitexin against hyperglycemia-related vascular complications via p38 MAPK/NF-κB inhibition.

## Introduction

Diabetes mellitus (DM) affected 537 million adults worldwide and was responsible for 6.7 million deaths in 2021. The International Diabetes Federation (IDF) predicts that the global prevalence of diabetes will increase to 643 million by 2030 and 783 million by 2045.^1^ Diabetes severely endangers public health and has reached an alarming level. High blood glucose (hyperglycemia) from diabetes causes severe damage to large (macrovascular) and small (microvascular) blood vessels and is the most severe complication of the disease. For example, diabetic nephropathy and retinopathy are the most significant contributors to end-stage renal disease and blindness, whereas arteriosclerosis is the leading cause of death in patients with diabetes.^2^ Therefore, it is crucial to develop effective vascular protection strategies for patients with diabetes.

Endothelial cells (ECs) form a monolayer lining of the blood vessel wall and maintain cardiovascular homeostasis by regulating vascular tone, blood fluidity, clotting and fibrinolysis, and smooth muscle cell proliferation. Endothelial dysfunction characterized by diminished nitric oxide (NO) production or availability is a critical pathological step in developing vascular complications in diabetes. The endothelial dysfunction should more appropriately be considered endothelial activation, a phenotype change involving the host defense response.^3^ The exposure of ECs to high glucose (HG) closely mimics that of inflammatory initiators and consequently activates NF-κB signaling.^4^ NF-κB activation promotes the expression of pro-inflammatory events and turns quiescent ECs into an activated state, which allows them to participate in the inflammatory response.^5,6^ Endothelial dysfunction is accompanied by chronic inflammation and contributes to the progression of diabetic vascular complications via various mechanisms. Abrogating the endothelial dysfunction and inflammation induced by hyperglycemia is clinically relevant. Inflammatory processes in endothelial cells directly contribute to endothelial dysfunction by increasing oxidative stress damage and reduces NO bioavailability, leading to impaired vasodilation and endothelial dysfunction.^7^

Vitexin (apigenin-8-C-β-glucopyranoside), referred to as “Mujingsu” in Chinese, is ubiquitously available in various medical plants such as hawthorn, gaillardia, Passiflora, bamboo, and beetroot. Due to its reliable safety and various biological activities, vitexin has received significant attention and is regarded as a potential therapeutic candidate for many diseases. Studies published by different groups have shown that vitexin possesses many pharmacological effects, including antioxidative and anti-inflammation properties, cardiovascular-, neuro-, and hepato-protective effects, anticancer and antidiabetes activities, adipogenesis suppression, treating nicotine addiction, and promoting hair growth. Vitexin has also been found to protect β-pancreatic cells from HG-induced oxidative injuries by scavenging free radicals and activating antioxidant enzymes, consequently enhancing glucose-stimulated insulin secretion and modulating the blood glucose level.^8^ Vitexin also decreases apoptosis and oxidant stress in human umbilical vein endothelial cells (HUVECs) by activating the Wnt/b-catenin and Nrf2 signaling pathways.^9^ Vitexin has significant antioxidant properties by scavenging free radicals and reducing oxidative stress. This is critical in protecting endothelial cells from oxidative injury, which is a critical factor in the development of atherosclerosis.^10^ These studies indicated that vitexin could be used as a potential therapeutic agent for diabetes, as well as in the prevention of diabetic vascular complications. However, its role in HG-induced endothelial cell dysfunction and inflammation has yet to be investigated. Therefore, this study aimed to examine whether vitexin protects HUVECs from HG-caused inflammatory responses.

## Materials and Methods

### Cell Culture and Treatment

Human umbilical vein endothelial cells (HUVECs) were purchased from the Bioresource Collection and Research Center (BCRC; Hsinchu, Taiwan). The cells were seeded onto culture dishes coated with 1% gelatin with medium 199 containing 25 U/ml heparin, 10% fetal bovine serum (FBS), 100 U/mL penicillin/streptomycin, and 30 μg/mL endothelial cell growth supplement (ECGS) at 37 °C with 5% CO_2_. Experiments were carried out with cells at passages 2 to 8. The culture medium and supplement were purchased from Gibco (Thermo Fisher Scientific, Waltham, MA, USA). The HUVECs were stimulated with 30 mM glucose (Sigma-Aldrich, St. Louis, MO, USA) with or without vitexin treatment. The human monocytic leukemia cell line (THP-1) was obtained from the ATCC. THP-1 cells were maintained in RPMI-1640 medium supplemented with 10% fetal bovine serum (FBS), 1% penicillin-streptomycin, 2 mM l-glutamine, and 0.05 mM 2-mercaptoethanol. Cells were cultured at 37 °C in a humidified atmosphere containing 5% CO_2_. Vitexin, BAY-985 (IκB/IKK inhibitor), ERK inhibitor (PD98059) and SB203880 (p38 MAPK inhibitor) were bought from Sigma-Aldrich and used for the experiments. Vitexin and inhibitors are dissolved in DMSO (1000× stock) and diluted to working concentrations fleshly during experiments.

### Western Blotting

RIPA buffer containing protease/phosphatase inhibitors (Thermo Fisher Scientific) was used to extract the proteins, and a BCA kit (Visual Protein, Taipei, Taiwan) was used to determine the protein concentration. SDS-PAGE was used to separate equal amounts of protein, which were then electroblotted onto the PVDF membranes. After blocking nonspecific binding via 5% skimmed milk, the membranes were incubated overnight with primary antibodies at 4 °C. The next day, the membranes were incubated with HRP-conjugated secondary antibodies for 1 h at room temperature and visualized using Clarity Western ECL Substrate (Bio-Rad, Hercules, CA, USA). The antibodies used in this study were anti-p-p38, anti-p38, anti-ERK, anti-p-ERK and p-I-κBα, which were bought from cell signaling; and p-IKKα and β-actin, bought from Thermo Fisher.

### NF-κB Activation Assays

The activation of NF-κB was determined using a TransAM DNA-binding ELISA kit according to the manufacturer’s instructions (Active Motif, Carlsbad, CA). Briefly, 5 μg of the nuclear extract was added to a 96-well plate precoated with an oligonucleotide containing the NF-κB consensus sequence. The activated p65 binding to this nucleotide in the extracts was detected using secondary antibodies conjugated to HRP. The colorimetric reaction was measured using a microplate reader (Bio-Rad) at 450 nm.

### Enzyme-Linked Immunosorbent Assay (ELISA)

The ELISA for the quantitative determination of human IL-6, IL-8, and IL-1β was performed on the stored supernatants from culture media, according to the manufacturer’s instructions (Elabscience, Houston, TX, USA) with a detection limit of 0.94 pg/mL for IL-6, 9.38 pg/mL for IL-8, and 0.39 pg/mL for IL-1β.

### Adhesion Molecule Expression Assay Using Flow Cytometry

After the treatment of HG with or without vitexin, cells were harvested and washed with PBS. Next, the cells were resuspended at a concentration of 1 × 10^6^ cells/mL in a flow cytometry buffer. Then, 100 μL of cell suspension was transferred to a tube, and 10 μL of antibody was used for the flow cytometry (ICAM-1, VCAM-1, E-selectin, and MCP-1). The cells were mixed and incubated in the dark at room temperature for 30 min. The cells were then washed with PBS twice. The fluorescence intensity was investigated to quantify the level of adhesion molecule expression.

### Monocyte Adhesion Assay

The HUVECs were seeded and incubated until they reached 90% confluence. THP-1 human monocytic cells were labeled with Cell Tracker dye (Thermo Fisher Scientific) for 30 min at 37 °C and seeded onto HUVECs. Nonadherent cells were washed after coculturing the HUVECs with the labeled THP-1 cells for 1 h at 37 °C. Triton X-100 (0.25%) with PBS was used to lyse the adherent THP-1 cells. The fluorescence intensity was investigated at 485 nm (excitation) and 538 nm (emission).

### Permeability Assay

Endothelial permeability was measured via the Costar Transwell system (Corning Inc., Corning, NY, USA) with FITC-labeled dextran tracers (AAT Bioquest, Pleasanton, CA, USA). HUVECs were cultured in a complete medium on top of 24-well Transwell inserts coated with gelatin to form a monolayer. In the last hour of the respective treatments, equal amounts of FITC-labeled dextran were added to the upper chamber and incubated for 2 h. The fluorescence in the lower compartment was measured by using a fluorometer. The permeability index was calculated using the tracer’s concentration in the lower and the upper chambers.

### Statistical Analysis

Data are shown as the mean value ± standard deviation (SD), and the analyses were performed using GraphPad Prism (GraphPad Software, La Jolla, CA, USA). The differences between the experimental groups were determined using Student’s *t* test and one-way analysis of variance (ANOVA). Statistical significance was set at *P* < 0.05.

## Results

### Vitexin Interferes with HG-Induced p38 MAPK and ERK Phosphorylation in HUVECs

**Figure 1:**
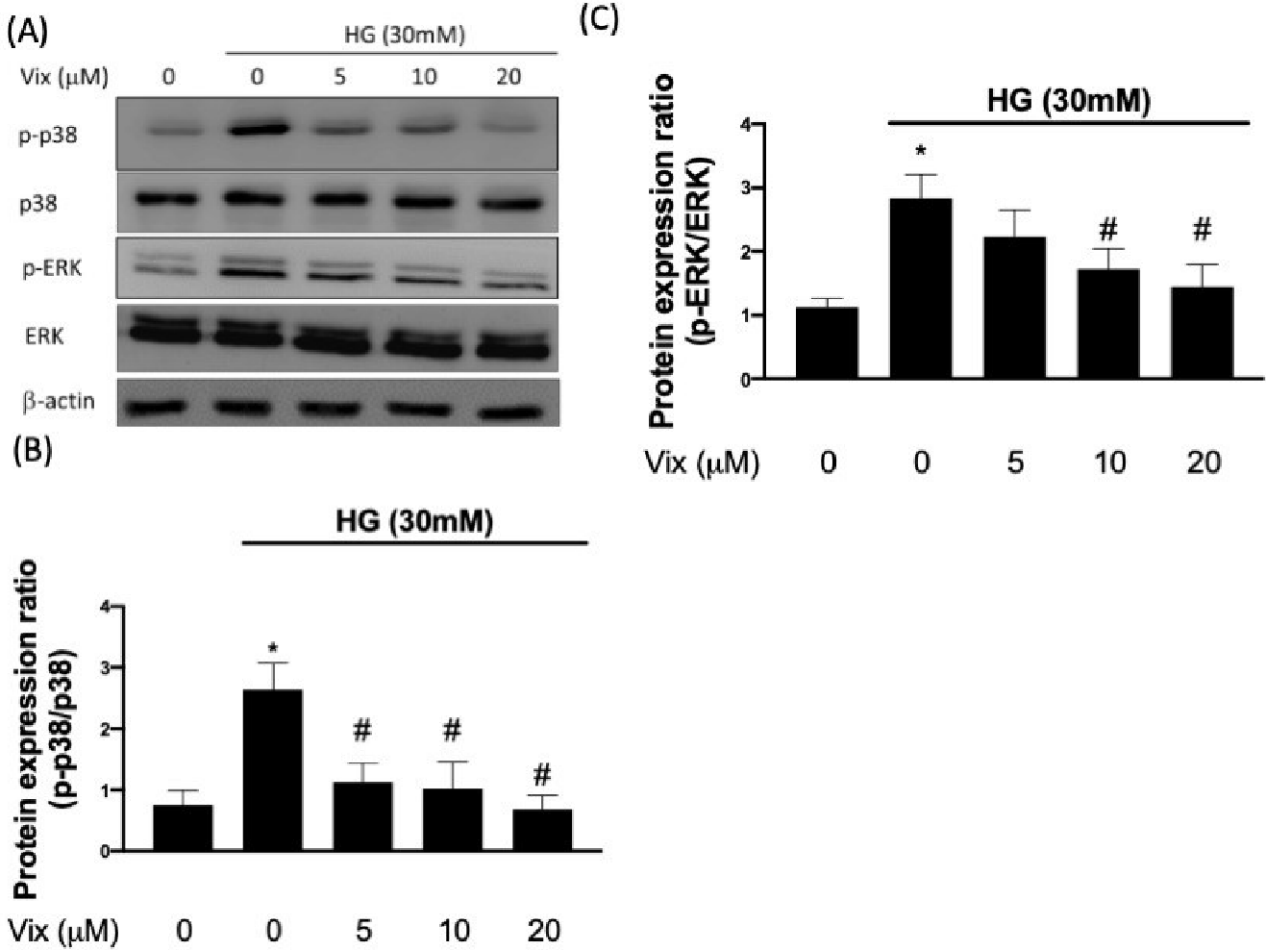
Vitexin suppresses HG-increased p38 phosphorylation. Representative Western blot images (A) and relative densitometric bar graphs of p-p38/p38 (B) and p-ERK/ERK (C) in endothelial cells stimulated by HG for 24 h with or without vitexin. (* indicates *p* < 0.05 compared with the control group; # indicates *p* < 0.05 compared to HG-stimulated cells.).

The MAPK pathway play critical roles in HG-caused endothelial dysfunction.^11^ For example, p38 mitogen-activated protein kinase (MAPK) plays a chief role in the response of endothelial cells to exogenous and endogenous stimulations.^12^ It was found that p38 MAPK and NF-κB collaborate to induce the expression of adhesion molecules and chemokines in HUVECs.^13^ In addition, it upregulates the ERK phosphorylation, contributing to differences in cardiovascular pathophysiology. ERK actives the proliferation and migration of vascular smooth muscle cell, leading to endothelia damage and subsequent cardiovascular complications.^14^ As shown in Figure 1, stimulated HUVECs with 30 mM glucose markedly upregulated the expression levels of phosphorylated p38 and ERK (*p* < 0.05). The HG-induced phosphorylation of p38 MAPK in the HUVECs was significantly interfered with by vitexin at concentrations from 5 μM to 20 μM (*p* < 0.05). The HG-induced phosphorylation of ERK in the HUVECs was significantly mitigated with by 10 μM to 20 μM (*p* < 0.05) vitexin.

### Vitexin Inhibits HG-Induced NF-κB Activation in HUVECs

**Figure 2:**
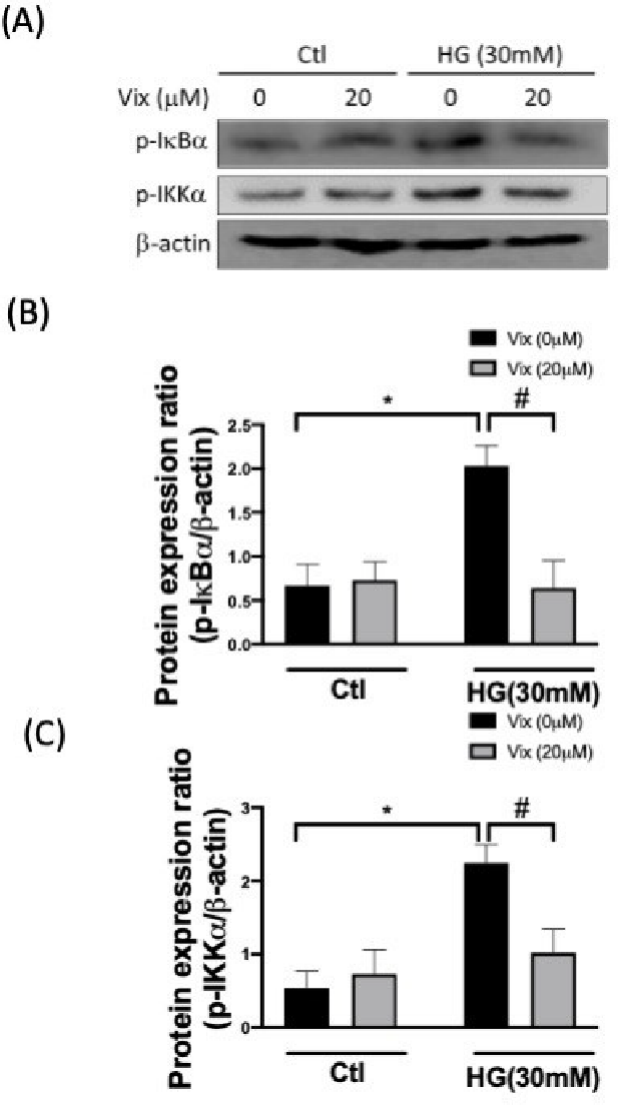
Vitexin mitigates HG-increased IκBα and IKKα phosphorylation. Representative Western blot images and relative densitometric bar graphs of phos-IκBα/β-actin and phos-IKKα/β-actin (A, B, C) in endothelial cells stimulated by HG for 24 h with or without vitexin. (* indicates *p* < 0.05 compared with the control group; # indicates *p* < 0.05 compared to HG-stimulated cells.).

**Figure 3:**
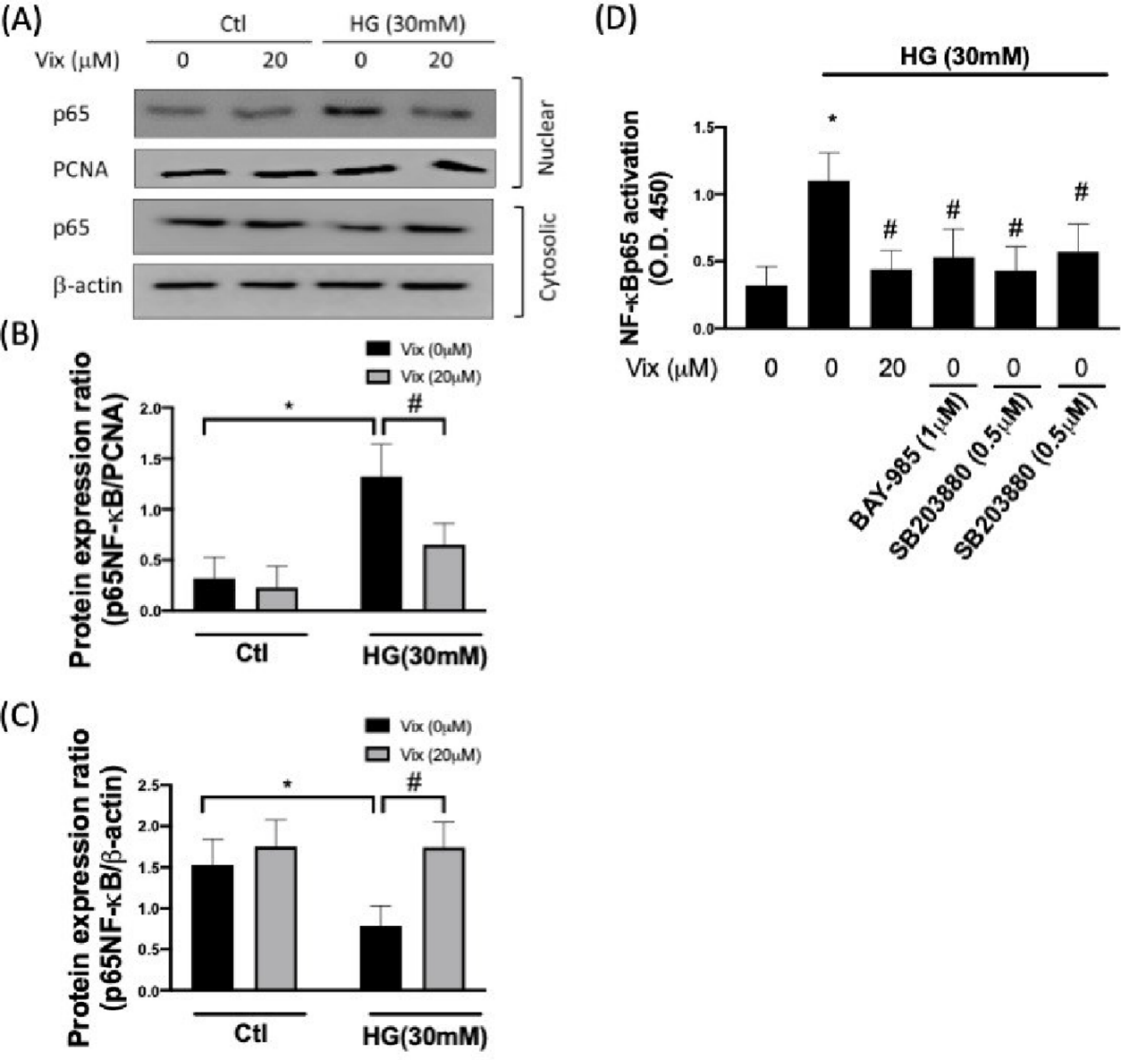
Vitexin attenuates HG-promoted NF-κB activity. Representative Western blot images and relative densitometric bar graphs of NF-κBp65/PCNA (nuclear fraction) and NF-κBp65/β-actin (cytosolic fraction) (A, B, C) in endothelial cells stimulated to HG for 24 h with or without vitexin. NF-κB activity was studied using an NF-κB activity assay kit (D). (* indicates *p* < 0.05 compared with the control group; # indicates *p* < 0.05 compared to HG-stimulated cells.).

We next assessed the effect of vitexin on the activity of NF-κB. Stimulated HUVECs with 30 mM glucose caused NF-κB activation via the phosphorylation of I-κBα and IKKα (Figure 2), leading to NF-κB p65 nuclear translocation (Figure 3A-C). The HG-induced phosphorylation of IκBα and IKK was blocked by vitexin (*p* < 0.05) (Figure 2). We observed that HG decreased cytosolic and increased nuclear NF-κB p65; such NF-κB p65 translocation was prevented by vitexin (*p* < 0.05) (Figure 3A-C). A NF-κB activity assay also demonstrated consistent results, vitexin significantly suppressed the HG-induced activity of NF-κB (*p* < 0.05) (Figure 3D). BAY-985 (I-κB/IKK inhibitor), SB203880 (p38 MAPK inhibitor) and PD98059 (ERK inhibitor) showed similar effects as vitexin on HUVECs (*p* < 0.05). These data suggest that vitexin effectively inhibits HG-induced NF-κB activation via the canonical signaling pathway.

### Vitexin Diminishes HG-Induced Transcription of Adhesion Molecules and Chemotactic Cytokine in HUVECs

**Figure 4:**
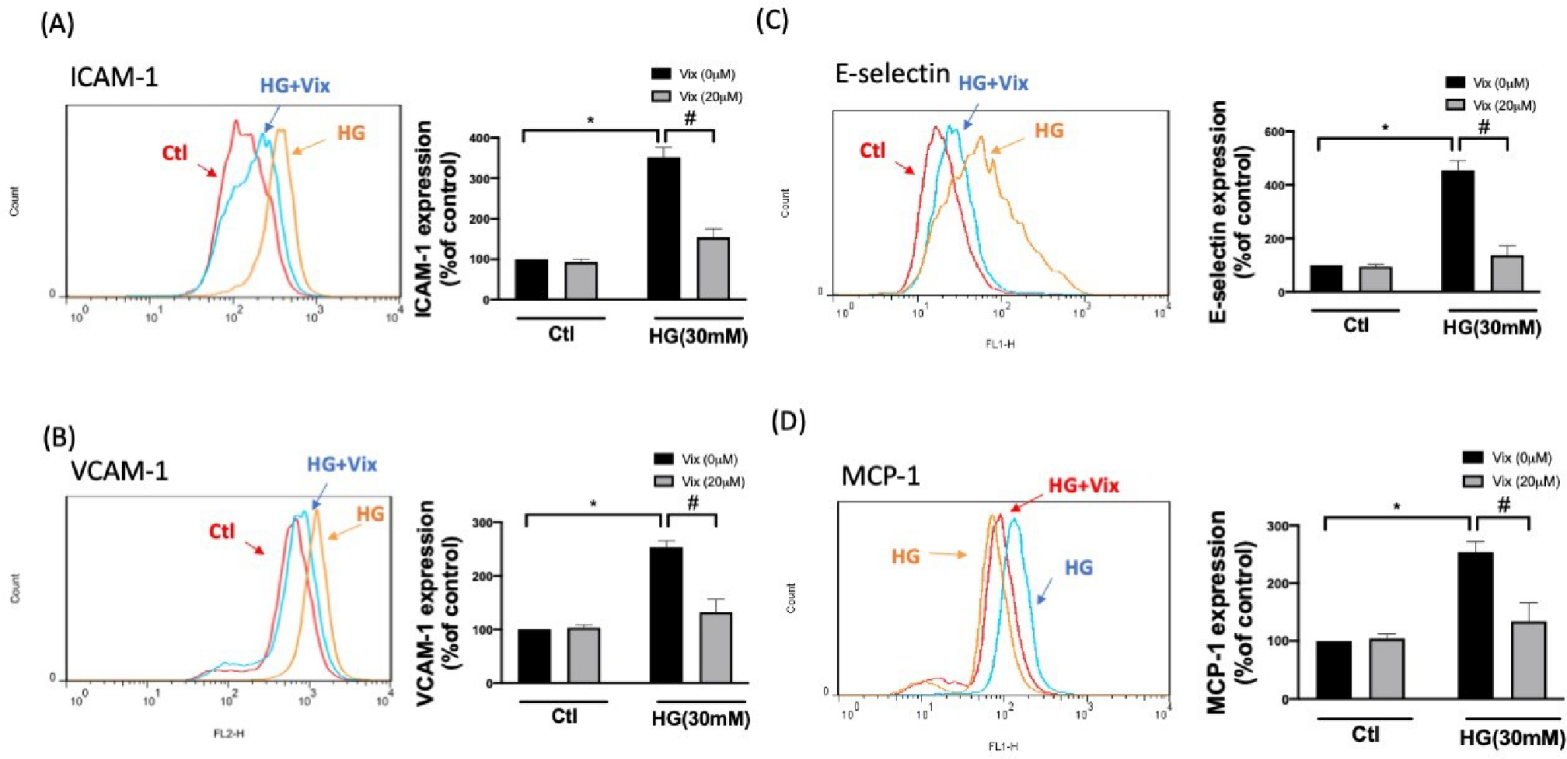
Vitexin inhibits HG-upregulated adhesion molecules. Cell surface expression of ICAM-1 (A), VCAM-1(B), E-selectin (C) and MCP-1 (D) was determined using flow cytometry. Both histogram and quantify results are presented. The values represent means ± SEM from three separate experiments. (* indicates *p* < 0.05 compared with the control group; # indicates *p* < 0.05 compared to HG-stimulated cells.).

The activated ECs express selectin and Ig-supergene family glycoproteins, resulting in rolling-firm adhesion and transmigration of leukocytes. These cell adhesion molecules mediate blood cell–endothelial cell interactions and guide the inflammatory response.^15^ Therefore, we performed real-time PCR to determine vitexin’s effect on the NF-κB mediation of adhesion molecule induction. HG promoted transcriptions of intercellular adhesion molecule 1 (ICAM-1), vascular cell adhesion protein 1 (VCAM-1), and E-selectin in the HUVECs. Vitexin significantly limited HG-induced increases in the transcription of these adhesion molecules (*p* < 0.05) (Figure 4A-C). The secretion of chemotactic cytokines causes chemotaxis and recruitment of leukocytes to the inflamed site. As shown in Figure 4D, vitexin also significantly restrained the HG-induced transcription of monocyte chemoattractant protein-1 (MCP-1) (*p* < 0.05), indicating that vitexin diminishes HG-associated chemotaxis.

### Vitexin Suppresses HG-Induced Secretion of Interleukins in HUVECs

**Figure 5:**
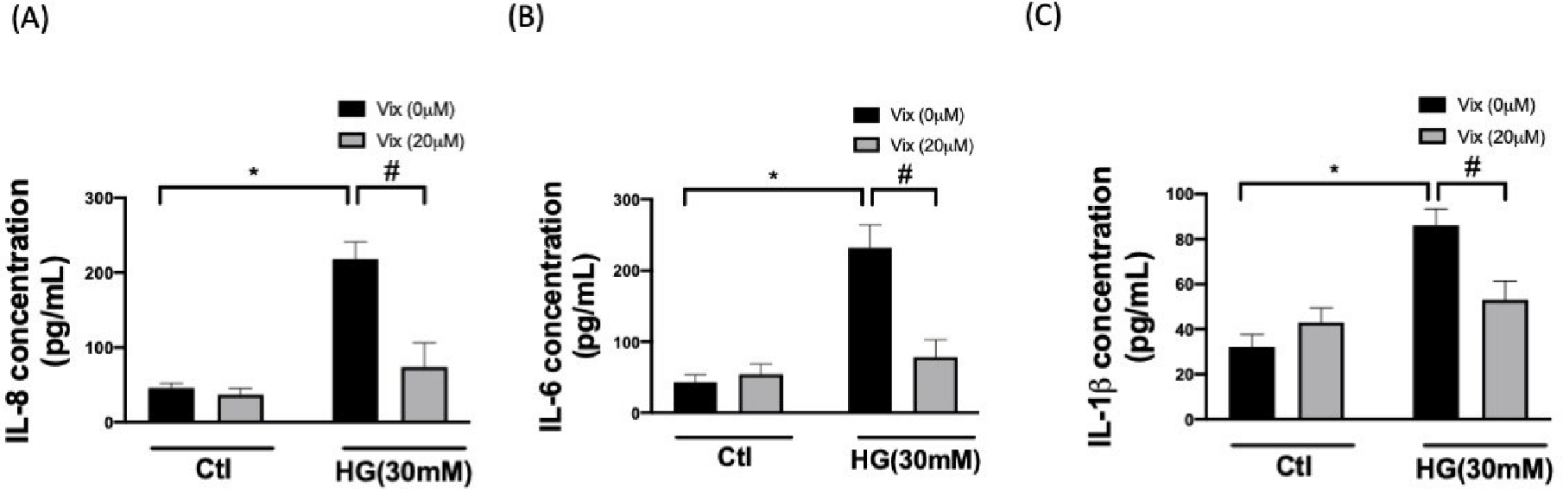
Vitexin mitigates pro-inflammatory cytokine release under HG stimulation. This figure illustrates the relative levels of key pro-inflammatory cytokines such as IL-8 (A), IL-6 (B), and IL-1β (C) using ELISA. (* indicates *p* < 0.05 compared with the control group; # indicates *p* < 0.05 compared to HG-stimulated cells.).

The role of interleukin (IL) in EC activation and vascular inflammation has been underlined.^16^ An ELISA demonstrated that secretions of IL-8, IL-6, and IL-1β were significantly decreased in HG-stimulated HUVCEs, and these protein levels were restored considerably by vitexin (*p* < 0.05) (Figure 5A-C). These results indicate that vitexin attenuates the endothelial inflammatory response induced by pathogenic mediators, such as hyperglycemia.

### Vitexin Limits the Permeability and Monocyte Adhesion of HG-Stimulated HUVECs

**Figure 6:**
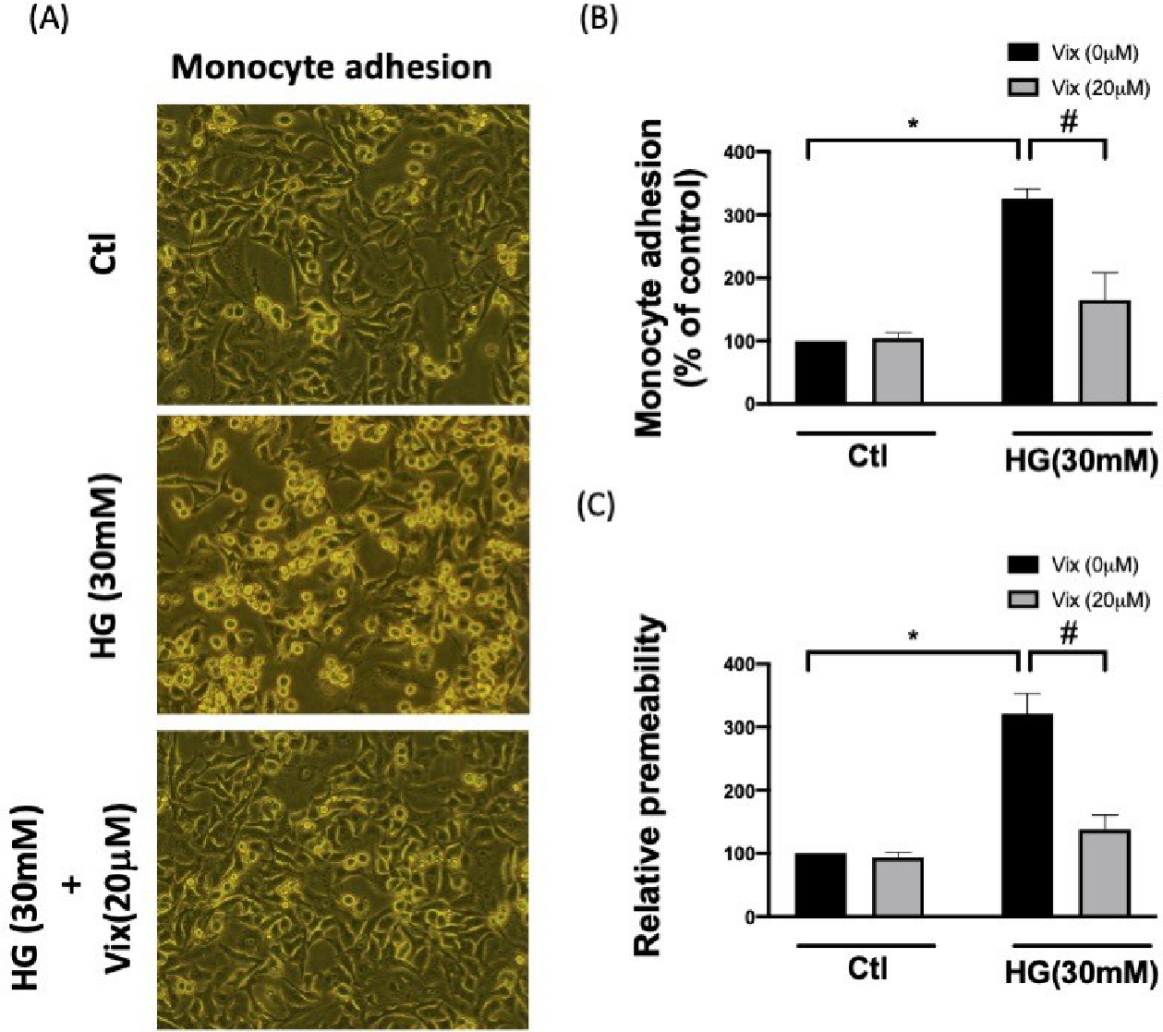
Vitexin protects against HG-enhanced monocyte adhesion and endothelial migration. This figure showcases the impact of HG exposure on endothelial injury, leading to enriched monocyte adhesion and endothelial migration. Monocyte adhesion to the damaged endothelial surface caused by HG stimulation with or without vitexin intervention (A). The Costar Transwell system (B) determined the endothelial migration rate under various HG stimulations. (C) The protective effect of vitexin in HG-caused vascular permeability. (* indicates *p* < 0.05 compared with the control group; # indicates *p* < 0.05 compared to HG-stimulated cells.)

As vitexin effectively inhibited HG-induced MAPK/NF-κB activation and diminished the expression of adhesion molecules, chemotactic cytokines, and pro-inflammatory cytokines, we hypothesized that vitexin would regulate EC permeability and monocyte adhesion. As expected, HG resulted in an increase in the adhesion of THP-1 monocyte to the HUVEC monolayer (*p* < 0.05) (Figure 6A-B), and vascular permeability (*p* < 0.05) (Figure 6C) was markedly reduced by vitexin. These results indicate that vitexin prevents vascular leakage and leukocyte recruitment. Vitexin moderates HG-induced type II endothelial activation in HUVECs through MAPK/NF-κB inhibition.

### Discussion

In healthy individuals, ECs are exposed to circulating blood glucose levels within a narrow range from 3.9 to 8.9 mmol/L throughout the day.^17^ In physiological conditions, ECs are quiescent and display minimal or absent proliferation, migration, and vascular leakage.^18^ These quiescent ECs prevent interaction with leukocytes by repressing the transcription of adhesion molecules and sequestering leukocyte-interactive proteins in secretory vesicles called Weibel–Palade bodies.^4,19^ Conversely, ECs undergo a phenotypic conversion, termed endothelial activation, resulting from stimulation from cytokines, adipocytokines, endotoxins, thrombin, antibodies, and other components as well as from mechanical stimuli. Endothelial activation was later grouped into two stages. Type I activation, an immediate transient process, involves remodeling endothelial cell–cell junctions to enhance vascular permeability and does not require de novo protein synthesis.^20,21^ The activation of pro-inflammatory factors such as NF-κB drives slow and sustained type II activation that involves the gene transcription and protein expression of adhesion molecules, cytokines, chemokines, and procoagulant factors.^20,22^ Following this, vascular leakage and leukocyte transmigration occur. In this present study, we demonstrate that vitexin effectively interferes with HG-induced phosphorylation of p38 MAPK and ERK, and significantly blocks HG-induced activation of the NF-κB, adhesion molecules, and monocyte adhesion in HUVECs.

Acute and long-term hyperglycemia has been shown to switch quiescent endothelial cells to an active state, eventually impairing endothelial function in both macrovascular and microvascular cells. Hyperglycemia impairs NO bioavailability and increases reactive oxygen species (ROS) production, thereby inducing endothelial injury and apoptosis.^9,23^ In the present study, we observed that the exposure of HUVCEs to HG increased vascular permeability (Figure 6C), promoted the transcription of ICAM1, VCAM1, and E-selectin (Figure 4A–C), and elevated the production of secretions of IL-8, IL-6, and IL-1β (Figure 5A–C). Our results agree with those of previous studies conducted by other groups in that hyperglycemia induces endothelial cell dysfunction at multiple stages, creates pro-inflammatory responses, and then leads to endothelial dysfunction and vascular complications.

Natural antidiabetic products have received increasing attention due to their significant biological activities and minimal side effects. The consideration of vitexin as a drug candidate for diabetes and related complications is a growing area of research among many scientists worldwide. Its use inhibits α-glycosidase activity and reduces postprandial blood glucose levels.^24^ In streptozotocin (STZ)-induced diabetic rats, vitexin exhibits protective effects via alleviating GPX4-mediated ferroptosis and improved spatial learning and memory retention via increases in superoxide dismutase (SOD) and glutathione peroxidase (GPx).^25^ Studies of the vascular protection effect and underlying mechanism of vitexin in diabetes remain limited. Zhang et al. demonstrated that vitexin protected HUVECs from high-glucose-induced injury by regulating Wnt/β-catenin-mediated apoptosis and Nrf2-mediated oxidative stress.^9^ In the present study, we demonstrated for the first time that vitexin prevents high-glucose-induced endothelial activation and alleviates vascular inflammation.

Chronic hyperglycemia exposure causes the accumulation of advanced glycation end products (AGEs). AGEs act on RAGE (receptor for AGEs) and activate NF-κB, thus promoting the transcription of its targeted genes.^6^ NF-κB activation enhances the expression of platelet-derived growth factor (PDGF), vascular endothelial growth factor (VEGF), transforming growth factor-β (TGF-β), and endothelin-1 (ET-1), contributing to vascular cell damage and angiogenesis.^26^ Excessive NF-κβ activation also triggers the calcification of endothelial cells, leading to the hardening of the medial layer of blood vessels.^27^ Most importantly, NF-κB mediates the production of pro-inflammatory responses, enhancing the secretion of cytokines, chemotactic factors, and adhesion molecules that accelerate the inflammatory process.^6,19,26^ The inhibition of NF-κB activation by expressing transdominant mutants of I-κB or dominant-negative versions of IKK in ECs blocks endothelial activation by suppressing the expression of chemokines and adhesion molecules.^28^ NF-κB is a promising target for the treatment of vascular complications in diabetes, particularly for the inhibition of NF-κB-mediated pro-inflammatory events. Our results identified vitexin as a potent NF-κB inhibitor (Figure 2 and 3) that can suppress both type I and type II endothelial activation, evidenced by the suppression of inflammatory cytokines, chemokines, and cell adhesion molecules and diminished permeability and monocyte adhesion in vitexin-treated HUVECs (Figures 4–6).

Endothelial activation is considered to be the initial step in the pathogenesis of vascular complications. Increased vascular permeability and the resultant vascular leakage are some of the first observable alterations in diabetic retinopathy and strongly correlate with vision impairment.^29^ Leukocyte adhesion to the endothelium is highly correlated to atheromatous plaque formation and initiates atherosclerosis development.^30^ The overexpression of MCP-1 results in macrophage accumulation and atherosclerosis acceleration, while the inhibition of MCP-1 alleviates lipid deposition and the atherosclerotic process. In addition to NF-κB, p38 MAPK is pivotal to adverse effects in hyperglycemia from diabetes. The activation of p38 MPAK protects ECs from the ROS-induced fragmentation of F-actin via actin remodeling into stress fibers. This may increase endothelial permeability, induce the inflammatory process, and lead to endothelial dysfunction.^12,31^ Furthermore, the activation of the p38 MPAK pathway accelerates VCAM-1 and MCP-1 expression in vascular endothelial cells via NF-κB-dependent and -independent signaling pathways.^12,13^ The up-regulated ERK phosphorylation in endothelial cells under hyperglycemic stimulations alters endothelial cellular functions, such as increased permeability, reduced nitric oxide production, as well as elevated expression of adhesion molecules.^32^ These events lead to endothelial damages, thereby providing an environment conducive to inflammation and formation of thrombosis.^33^ As shown in our results, vitexin is a potential inhibitor of p38 MAPK and ERK (Figure 1). Thus, vitexin prevents high-glucose-induced endothelial activation and alleviates vascular inflammation via multiple signaling pathways.

The limitation of this presented study is the exclusive use of in vitro techniques. While in vitro studies provide valuable insights into cellular mechanisms and responses under controlled conditions, the results still cannot fully replicate the complexity of physiology in human. Thus, animal study will be our major direction in future study. However, our study demonstrated that vitexin protected HUVECs from HG-induced endothelial dysfunction and inflammation via the suppression of the NF-κB signaling pathway, suggesting that vitexin might serve as a potential drug for vascular complications of diabetes.

## Data Availability

The data supporting this study’s findings are available from the corresponding author upon reasonable request (kunlingtsai@gmail.com).
